# Validation of LDLr Activity as a Tool to Improve Genetic Diagnosis of Familial Hypercholesterolemia: A Retrospective on Functional Characterization of LDLr Variants

**DOI:** 10.3390/ijms19061676

**Published:** 2018-06-05

**Authors:** Asier Benito-Vicente, Kepa B. Uribe, Shifa Jebari, Unai Galicia-Garcia, Helena Ostolaza, Cesar Martin

**Affiliations:** Instituto Biofisika (UPV/EHU, CSIC) and Departamento de Bioquímica, Universidad del País Vasco, Apdo. 644, 48080 Bilbao, Spain; asierbenitovicente@gmail.com (A.B.-V.); kepa1985@gmail.com (K.B.U.); shifajebari@gmail.com (S.J.); u.galiciag@gmail.com (U.G.-G.); elenaamaya.ostolaza@ehu.eus (H.O.)

**Keywords:** Low Density Lipoprotein receptor (LDLr), variants, familial hypercholesterolemia, is silico, in vitro, functional validation

## Abstract

Familial hypercholesterolemia (FH) is an autosomal dominant disorder characterized by high blood-cholesterol levels mostly caused by mutations in the low-density lipoprotein receptor (LDLr). With a prevalence as high as 1/200 in some populations, genetic screening for pathogenic LDLr mutations is a cost-effective approach in families classified as ‘definite’ or ‘probable’ FH and can help to early diagnosis. However, with over 2000 LDLr variants identified, distinguishing pathogenic mutations from benign mutations is a long-standing challenge in the field. In 1998, the World Health Organization (WHO) highlighted the importance of improving the diagnosis and prognosis of FH patients thus, identifying LDLr pathogenic variants is a longstanding challenge to provide an accurate genetic diagnosis and personalized treatments. In recent years, accessible methodologies have been developed to assess LDLr activity in vitro, providing experimental reproducibility between laboratories all over the world that ensures rigorous analysis of all functional studies. In this review we present a broad spectrum of functionally characterized missense LDLr variants identified in patients with FH, which is mandatory for a definite diagnosis of FH.

## 1. Familial Hypercholesterolemia (FH)

Familial hypercholesterolemia (FH) is one of the most frequent dyslipidaemias characterized by high concentrations of total and LDL cholesterol (LDL-c) leading to accelerated atherosclerosis and premature coronary heart disease (CHD) [1,2]. FH is an autosomal monogenic disorder and, with a frequency estimated between 1:200–1:250, constitutes one of the most serious com­monly inherited metabolic diseases. Despite its high prevalence, FH is still severely underdiagnosed and undertreated. Autosomal dominant mutations in *LDLR* (encoding the LDL receptor), *APOB* (encoding apolipoprotein B100 (apoB100)), and *PCSK9* (encoding proprotein convertase subtilisin/kexin type 9) genes account for most cases of FH [3,4,5,6]. Most individuals with FH are heterozygous for mutations in one of these genes and, therefore, have heterozygous FH. Being its penetrance almost 100%, half of the offspring (mean prevalence) of an affected parent have a severely increased plasma cholesterol level from birth onwards. Mutations in *LDLR* are the main genetic cause of FH [7,8,9] constituting more than 90% of the mutations found in FH patients, with more than 2000 *LDLR* genetic variants submitted so far to the Human Gene Mutation Database (HGMD).

## 2. FH Diagnosis

At present, there are neither conclusive clinical criteria for the diagnosis of FH nor standardized processes for phenotypic diagnosis [10]. The clinical and biological diagnosis of FH is frequently based on the Dutch Lipid Clinic Network (DLCN) score, which requires a detailed family history and physical stigmata [11]. Therefore, clinical criteria used to identify patients with FH include high plasma levels of total and LDL-C (250 mg/dL or 7 mmol/L), family history of premature coronary artery disease (CAD), tendon xanthomata, corneal arcus, and elevated LDL cholesterol [12,13,14]. In addition to DCLN score, Simon-Broome and US MedPed are also widely used. The best diagnostic approach in most populations is to measure LDL-C levels in all first degree relatives of a FH proband and screen all second-degree family members [15]. Heterozygous FH individuals have LDL-C levels roughly 2 to 3 times higher than those in the general population, ranging from 190 to 400 mg/dL (4.9–10.3 mmol/L). Although clinical diagnostic criteria have been extensively used for FH [16], genetic testing is the preferred method for FH because it provides an unequivocal diagnosis [8,17,18] and it also provides information for family cascade screening. However, correctly interpreting the clinical significance of LDLr variants continues to be a constant challenge for molecular diagnostic practice and clinical diagnosis can only be confirmed when a mutation is functionally characterized and proven to affect LDL metabolism. LDLr variants can be grouped into 2 categories: truncating and nontruncating variants. Truncating variants such as nonsense variants, out-of-frame indels, most splicing variants, and partial gene deletions are known to have a deleterious effect on the function of the LDLr protein and are considered to be pathogenic variants without the need of functional characterization [19]. Nontruncating variants consist of single or multiple nucleotide substitutions and in-frame indels. It is often more difficult to predict their pathogenicity. Currently, the procedure for functional validation has become widespread because new and cost-effective methodologies allow evaluation of these nontruncating variants through radioactive assays and fluorescence-based approaches. Therefore, functional validation of these variants can be performed both ex vivo and in vitro by using fluorescently labelled -antibodies or -LDL to determine LDLr activity in heterologous cell models to directly demonstrate disease causality.

## 3. LDL Receptor

**Figure 1:**
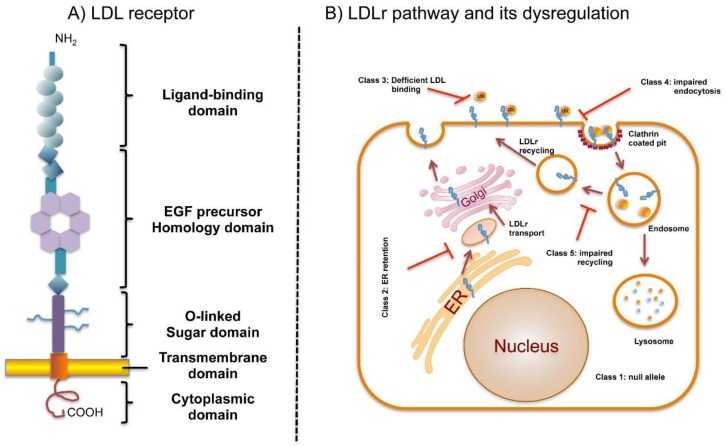
Domain organization of LDLr and LDLr pathway and its dysregulation by defective mutations. (**A**) Schematic representation of LDLr domains; (**B**) LDLr cycle. LDLr is synthesed at ER, transproted to Golgi where is further processed with glycosilations. Mature LDLr is transported to the plasma membrane, where the ligand-binding domain binds to the apo B100 moiety on LDL particles. The LDLr/LDL complex undergoes endocitosis and within the cell, LDL particle components are targeted for lysosomal degradation, whereas the LDLR is recycled to the cell surface. LDLr mutations affecting different LDLr cycle results in dysregulation of the cycle.

The mature LDLr is a type I transmembrane protein of 839 amino acids which regulates cholesterol homeostasis in mammalian cells [3]. LDLr is mapped to 19p13.1–13.3 on the short arm of chromosome 19, spans 45 kb and consists of 18 exons and 17 introns that are transcribed and translated into five distinct domains which form the cell-surface LDL receptor [20]. The protein is encoded as a precursor of 860 amino acids comprising a 21-residues signal sequence at the N-terminus that is excised during protein translocation into the endoplasmic reticulum (ER) [21]. LDLr is synthesized on ribosomes of ER, then folded and partially glycosylated within ER and finally matured in the Golgi complex, where glycosylation is completed [22]. The mature protein is structured into functional domains organized in an ectodomain and intracellular domain. The ectodomain is encoded by exons 2–15 and harbours a ligand-binding domain, an epidermal growth factor (EGF) precursor homology domain and a C-terminal domain enriched in O-linked oligosaccharides (Figure 1A).

The ligand binding domain contains 7 cysteine-rich repeats (LR1 to LR7) of approximately 40 amino acids with three disulphide bridges each (CysI-III, CysII-V, CysIV-VI). In addition, an acidic residues cluster coordinates a Ca^2+^ ion which is required for correct folding of the domain [23]. Binding of lipoproteins to the LDLr appears to be mediated by an interaction between acidic residues in the LDLr-binding domain and basic residues of apoE and apoB100 [24,25]. The intracellular release of the cargo is driven by a low-pH-induced conformational change of LDLr from an open to a closed conformation [23,26,27]. Binding to different ligands appears to require different subsets of LR modules [23,25,28]. The LR modules are interspaced by a short linker sequence mostly formed by four residues ending in Thr with the sequence motif XXC_6_XXX**TC**_1_-XX. Some linkers, however, are longer. It has been shown that O-glycans in the LR ligand-binding region of LDLr as well as VLDLr are important for high-affinity lipoprotein binding and uptake [29].

The EGF precursor homology domain participates in the acid-dependent lipoprotein release in the endosome and consists of two EGF-like domains, six YWTD repeats that form a six-bladed β-propeller, and a third EGF-like repeat [30,31]. It has been shown that PCSK9, a secreted glycoprotein, promotes degradation of the LDLr, thereby preventing clearance of LDL-C by the cells [32]. It also interacts with the EGF-A domain of the LDLr at the cell surface and binds to the full-length receptor with a much higher affinity in the acidic environment of the endosome. Consequently, the receptor is transported from the endosome to the lysosome for degradation, rather than being recycled [32].

The C-terminal domain enriched in O-linked oligosaccharides contains 58 amino acids rich in threonine and serine residues. It is thought that this domain plays a role in the stabilization of the receptor [8]. This region shows minimal sequence conservation among six species analyzed and can be deleted without adverse effects on receptor function in cultured fibroblasts [33].

The intracellular domains are encoded by Exons 16–17 and together constitute the transmembrane domain. The TM domain contains 22 hydrophobic amino acids that are essential for anchoring the LDLr to the cell membrane. The cytoplasmic domain of LDLr, consists of 50 amino acid residues and contains two sequence signals for targeting the LDLr to the cell surface and localizing the receptor to coated pits [34]. Internalization of the LDLR also requires this cytoplasmic domain [35,36].

LDLr transcription is tightly regulated by the sterol-responsive element binding protein-2 (SREBP-2) through a feedback mechanism that responds to variations in intracellular sterol concentrations and cellular demand for cholesterol [37]. In addition to classical transcription regulators, a class of noncoding RNAs termed microRNAs (miRNAs), has emerged as critical regulators of gene expression acting predominantly at the post-transcriptional level [38]. In particular, miR-148a directly controls LDLr activity and is transcriptionally activated by SREBP1c in vitro and in vivo [39].

## 4. LDLr Pathway and Its Dysregulation by Defective Mutations

Upon lipoprotein binding to LDLr at the cell surface, the complex is internalized through clathrin-coated pits into clathrin-coated vesicles [40] (Figure 1B). These vesicles fuse with early endosomes, and acidification of the endosomal pH promotes LDL release, which is later degraded in lysosomes. Normally, the LDLr is returned to the membrane and enters a new cycle [23].

This system maintains a constant level of cholesterol in hepatocytes and other cells by controlling both the rate of cholesterol uptake from LDL and the rate of cholesterol synthesis [41]. LDLr mutations affect different parts of this LDLr cycle leading to FH. LDLr mutations are thus classified depending on the phenotypic behaviour of the mutant protein (Figure 1B) [42,43]: Class 1. Synthesis alteration, known as “null alleles”; Class 2. Defective transport to Golgi or to the plasma membrane because the synthetized proteins do not have an adequate three-dimensional structure and are retained, completely or partially (2A and 2B, respectively) in the ER; Class 3. Deficient binding to ApoB, LDL binding activity is 2% to 30% of normal due to rearrangements in repeat cysteine residues in binding ligand domain or repeat deletions in EGFP-like domain; Class 4. Impaired endocytosis, LDLr is not recruited into clathrin-coated pits; Class 5. Alteration in the recycle mechanism as a consequence of an impaired LDL release in endosomes causing the receptor to be degraded in the lysosome. Recently, a sixth class of mutations in the LDLr that interfere with insertion of the LDLr into the cell membrane resulting in LDLr secretion has been described [44].

## 5. Determining the Pathogenicity of LDLr Variants

The majority of FH patients with positive genetic testing results have rare pathogenic variants in LDLr [45] which comprise 60% of the ~2000 LDLr genetic variants that have been submitted to the HGMD. Determining pathogenicity of LDLr is a key challenge in genomic medicine; therefore, several approaches including computer prediction algorithms, in vivo and in vitro experimental evidence, are used to gain information about variant effects [46].

### 5.1. In Silico Analysis

The revolution in DNA sequencing methodologies has tremendously increased the number of gene sequences over the last several years, and these technologies continue to evolve [47,48]. Next-generation sequencing (NGS) in combination with sequence target enrichment methods are useful in molecular diagnostics of FH [49]. Single-nucleotide polymorphisms (SNPs) are considered to be the most common genetic changes that result from alterations in a single nucleotide. Among SNPs, nonsynonymous SNPs (nsSNP) are associated with single amino acid substitution in the coding regions of a gene that may have a drastic effect on the structural and functional properties of the corresponding protein. These nsSNPs have been the subject of many recent studies and a large amount of data now exists in public repositories such as dbSNP [50], HGVBase [51] and HGMD [52]. To manage the large amount of data produced, computational tools to predict the functional effects of sequence variations are under constant development to prioritize high-risk variants that should be experimentally characterized for pathogenicity. These tools have been developed based on features such as amino acid or nucleotide conservation and biochemical properties of the amino acid substitutions. Identification of single nucleotide polymorphisms in the coding region of a gene that have implications in inherited human diseases is the fundamental objective of research in medical genetics. The most used pathogenicity predictor open access software to assess the effect of LDLr variants are PolyPhen-2 [53], Sorting Tolerant From Intolerant (SIFT) [54], Consensus Deleteriousness score of missense SNVs (Condel) [55], Mutation taster [56], Grantham Score [57] and PhyloP [58]. However, individual tools often disagree, in part because they utilize different predictive features. Understanding how amino acid substitutions affect protein functions is critical for the study of proteins and their implications in diseases. Another limitation is that prediction results are hard to interpret without physicochemical principles and biological knowledge. For this reason, there is a growing need for the development and evaluation of tools for predicting the pathogenicity of rare variants. Furthermore, functional validation of these LDLr variants must be conducted in order to identify which mutations lead to a functional loss of receptor activity.

### 5.2. Functional Characterization of LDLr Variants

**Figure 2:**
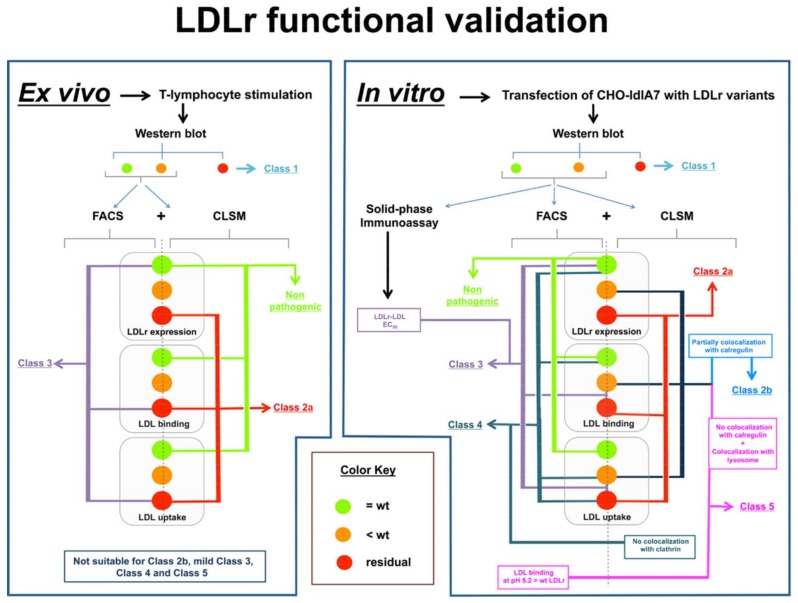
Flowchart of the used methodologies to functionally characterize LDLr variants ex vivo and in vitro. Functional studies of LDLr variants are mainly conducted using two major approaches: 1. ex vivo methods, using cells from Familial Hypercholesterolemia (FH) patients (left-hand panel); 2. in vitro methods using cell lines transfected with the LDLr variant (right-hand panel). LDLr activity determination is based in combination of different methodologies: Western blot to analyse LDLr expression followed by fluorescence-activated cell sorting (FACS) and Confocal Laser Scanning Microscopy (CLSM) that allow assessment of Class type mutation. The ex vivo approach is adequate for Class 1, Class 2a and Class 3 LDLr variants. In vitro characterization allows identification of Class 2b mutations by colocalizing the LDLr variants in the ER with calrgulin; using a solid-phase immunoassay it is possible to determine LDLr-LDL EC_50_ values for Class 3 mutations which is important to understand mild pathogenic variants; Class 4 variants are classified by complementing CLSM with a colocalization assay with clathrin and, identification of Class 5 mutants is performed by absence of LDLr colocalization with calregulin, LDLr colocalization with a lysosome marker complemented by a FACS analysis of LDL binding to LDLr at different pH (7.4–5.2).

Functional assays are a direct method to determine whether the activity of a mutant protein is altered by taking into account all the involved biological mechanisms. To date, functional studies of LDLr variants have been conducted using two major approaches: 1. ex vivo methods, using cells from FH patients; 2. in vitro methods using cell lines transfected with the LDLR mutant (Figure 2).

#### 5.2.1. Ex vivo Functional Validation

Since the first demonstration by Brown and Goldstein of the presence of a measurable LDL receptor pathway in cultured skin fibroblasts from FH-homozygotes and normolipidemic controls with ^125^I-labeled LDL [59], research has focused on the development of new and less invasive methodologies for LDLr activity assessment. New strategies that use lymphocytes allow validation of LDLr functionality by immortalization mediated by Epstein-Barr virus [60,61,62], stimulation of LDLr expression in lymphocytes by incubation with statins [63,64], or treatment of cells with mitogens or CD3/CD28 beads to stimulate T-lymphocytes [62,65]. Comparative studies between results in fibroblasts with those obtained from immortalized lymphoblastoid cells from the same patient showed similar results [66]. T-lymphocyte stimulation by CD3/CD28 beads followed by determining LDLr activity through fluorescence-activated cell sorting (FACS) is a simple strategy used for functional assays. This technique requires incubating cells from FH patients for 72 h with CD3/CD28 beads in a medium containing a lipoprotein deficient serum to upregulate the LDLr, and then they are incubated with labelled-LDL allowing the detection of the bound and/or internalized LDL amounts. Nowadays, LDL are normally labelled with fluorescent molecules that allow obtaining an accurate analysis by FACS [62,67]. Specifically, labelling LDLr with a fluorescent antibody or LDL with a fluorescent antibody allows determination of LDLr expression at the cell membrane and LDL-LDLr binding, respectively. LDL labelling is conducted by incubating cells for 4 h at 4 °C with fluorescent-labelled LDL [67,68]. A recent advance in determining LDL uptake was introduced by our group and consists of a combination of Fluorescein IsoTioCyanate labelled LDL (FITC-LDL) and Trypan-blue dye [67]. This method allows determination of LDL uptake in a single step because addition of Trypan blue to the cell suspension quenches external fluorescence from LDL bound to membrane receptors, allowing fluorescence quantification of internalized LDL exclusively [67]. Confocal microscopy with an anti-LDLR antibody is used to verify localization of the LDLR on the plasma membrane and of ApoB after endocytosis. The ex vivo approach is very suitable in assessment of Class 1, Class 2a and Class 3 LDLr variants in which LDL binding is highly impaired. However, ex vivo studies have the following limitations: because lymphocytes studies are from heterozygote patients, interference of the wild type allele has to be taken into account. Even in the presence of a null allele leading to a total absent protein, the activity of the LDLr synthesized from the normal allele is still detectable and the total measured activity is around 50%. If the variant under study is Class 2b, Class 3 (without a complete loss of binding capacity), Class 4 and Class 5 the activity data may range from 70–90% compared to activities of lymphocytes carrying wild type LDLr in both alleles. For this reason, ascertaining pathogenicity in these cases needs further analysis. Another limitation of ex vivo assays is that localization of LDLr to different subcellular compartments by confocal microscopy is difficult due to the extremely small cytoplasm of lymphocytes. Advantages and disadvantages between functional validation methodologies are shown in Table 1.

#### 5.2.2. In Vitro Functional Validation

In vitro cell line model systems are particularly useful to help further our understanding of the mechanisms underlying pathogenicity of LDLr variants. The use of cell line models to study LDLr variant activities has many advantages: cell lines represent a renewable resource, are well-controlled systems and there is no need for clinical samples. The analyses performed on these cell lines to functionally validate LDLr variants are similar to those performed on ex vivo assays, including the use of FACS measurements with antibodies [69] or fluorescently-labeled LDL and confocal microscopy using antibodies for ApoB or markers for the endoplasmic reticulum [69]. Cells are transfected with an expression plasmid in which the LDLr carrying the studied mutation is cloned. The methodology used for that purpose is as follows:

#### 5.2.2.1. Cell Transfection

LDLr-deficient Chinese hamster ovary (CHO) cell line ldlA7 (CHO-ldlA7) is transfected with plasmids carrying the LDLr variant of interest [70,71]. Different methods of transfection are suitable depending on laboratory experience. Transfected cells are maintained in culture during 48 h to achieve maximal LDLr expression.

#### 5.2.2.2. Western Blot Analysis

Usually the first step in functional characterization is to evaluate receptor expression. To do so, immunoblotting is used to test if the LDLr variant is able to go from the precursor to the mature form. Experimentally, cell lysates have to be prepared, protein concentration determined, and fractionated by electrophoresis. Then, for semiquantitative immunoblotting, proteins are transferred to nitrocellulose membranes which are immunostained using the appropriate antibodies. The signals are then developed and quantified. The relative LDLr expression for the LDLr variant is calculated as the ratio between the sum of band intensities corresponding to the mature and precursor form of LDLR protein to that of a constitutive protein such as GAPDH.

#### 5.2.2.3. Quantification of LDLr Expression by Flow Cytometry

To determine LDLR cell surface expression by FACS, transfected CHO-ldlA7 cells are incubated with a primary antibody anti-LDLR for 1 h at room temperature, then washed with PBS-1% BSA and incubated with secondary antibody Alexa Fluor 488-conjugated antimouse IgG. Fluorescence is then acquired through FACS and compared with fluorescence obtained in cells expressing wild type LDLr.

#### 5.2.2.4. Quantification of LDLR Activity by FACS

Transfected CHO-ldlA7 cells are incubated for 4 h at 37 °C or 4 °C with 20 μg/mL FITC–LDL to determine LDLr activity or LDL–LDLr binding, respectively. After incubation with FITC–LDL, CHO-ldlA7 cells are washed twice in PBS-1% BSA, fixed on 4% formaldehyde, and washed again. To determine the amount of internalized LDL, Trypan blue solution is added to a final concentration of 0.2% directly to the samples, eliminating the extracellular signal due to the noninternalized LDL–LDLr complexes.

#### 5.2.2.5. Confocal Laser Scanning Microscopy

Confocal laser scanning microscopy is used to analyze LDL-LDLr binding, uptake, expression of LDLr, and enables determination of Class type mutation by testing LDLr colocalization with clathrin, lysosomes, or endoplasmic reticulum (ER). Cells are plated in coverslips and transfected with the LDLr containing plasmids. After 48 h, nonlabelled lipoproteins are added and cells, further incubated for a 4 h then stained with the appropriate primary antibodies for 16 h at 4 °C followed by incubation with fluorescent secondary antibodies. Cells are then visualized using a confocal microscope and images processed and fluorescence intensities quantified.

#### 5.2.2.6. LDL–LDLr Binding at Different pH

To determine if the defect of an LDLr variant is due to defective LDLr recycling, an LDL binding assay is performed at different pH’s to mimic the acidification process occurring in the endosome after LDL endocytosis. To do this, transfected cells are incubated with 20 μg/mL of LDL–FITC for 30 min in a 0.4-M sucrose medium at different pH’s. Then, cells are washed three times to remove unbound LDL, fixed with 4% paraformaldehyde and the amount of bound LDL–FITC is quantified by FACS.

#### 5.2.2.7. LDLr-LDL Affinity Assessment

In order to better classify Class 3 mutations from mild to severe pathogenic effect, a modified ELISA binding assay with purified soluble wt LDLr (sLDLr) or and the variant of interest can be performed. sLDLr variants are coated in 96-well and incubated with freshly purified human LDL for 2 h at RT, then samples are incubated with anti-Apolipoprotein B for 1 h followed by peroxidase-conjugated IgG for 1 h, and developed with a chromogenic substrate. After photometric quantification, EC_50_ values are calculated providing information about LDLr affinity to LDL [29].

In the recent years, our group has been actively using fluorescent-based methodologies to characterize and classify LDLr variants; the obtained results to date are shown in Table 2.

## 6. ClinVar: Variant Pathogenicity Assignments based on LDLr Functional Characterization

The identification of many novel LDLr variants by NGS in clinical genetic testing has led to the need for storing data about variant classification in a clinically-applicable location. Thus, several general and gene-specific databases are available for use by investigators and clinicians, including the National Center for Biotechnology Information (NCBI) ClinVar database [77]. The ClinVar database at NCBI archives and aggregates submitted interpretations of the clinical and/or functional significance of variants for specified conditions, with opportunities to provide supporting evidence. Recently, the ClinVar database related to LDLr has been updated with variants stored in the LDLr—specific Leiden Open Variation Database (LOVD), increasing the size of the ClinVar LDLr database from 338 variants as of July 11, 2016, to 2248 variants as of April 30, 2018. Some research-oriented submissions may provide functional significance based on experimental evidence, which may inform the clinical interpretation of the same variant in patient encounters. To date there are 794 unique missense LDLr variants annotated in ClinVar identified by both research and clinical testing. Among them, the reported clinical significance is as follows: 2.02% benign/likely benign, 7.81 of uncertain significance, 62.80% pathogenic/ likely pathogenic and, 27.33% conflicting interpretations. The category “conflicting interpretations” includes variants with multiple submissions where the associated classifications were: benign/likely benign + uncertain significance; pathogenic/likely pathogenic + uncertain significance; or benign/likely benign + pathogenic/likely pathogenic.

As mentioned above, the most used methodologies to determine LDLr functionality ex vivo and in vitro are based on the use of radioactivity or fluorophores. LDL uptake and degradation of ^125^I-labeled LDL has been commonly used in radioactivity-based methods, a methodology that is being replaced by the use of fluorescent-labeled LDL and antibodies for determining activity of LDLr [20,65,67,78]. Both methods have been used indistinctly, probably depending on the research laboratory facilities or continuation of the previously methodology in a specific laboratory. To date, 794 unique missense pathogenic LDLr variants have been annotated in ClinVar. Only a minority of these 794 variants have been proven pathogenic. Among them, the activity of 100 has been experimentally characterized (Table 3). Radioactive techniques have been used to functionally characterize 62 LDLr variants and fluorescence-based methodologies to characterize 33 variants. Two mutations have been assessed by both methodologies and three variants were characterized by other techniques (Western blot and RNA studies). Although the number of characterized variants may seem low (13% of the annotated missense variants), extensive work is being done by multiple laboratories to characterize the remaining variants. In the next few years the percentage of the functionally characterized variants will increase notably. In this respect, the use of fluorescence methodologies is increasing the number of validated variants because FACS allows easier quantification of LDLr expression at the cell surface and LDL uptake provides a better characterization of the defect associated with each mutation. In fact, FACS complementation with confocal microscopy allows detection of the subcellular localization of the LDLr, which allows assignment of the class type of each variant studied [69].

## 7. Conclusions

Recent advances in genetic sequencing technology have resulted in remarkable improvements in the speed, throughput and identification of LDLr variants occurring in FH patients. To date, more than 2000 LDLr variants associated with FH have been described but only a minority of them have been functionally validated and proven to be the cause of the disease. Awareness and identification of the pathogenic variants causing FH would provide a definitive diagnosis. Additionally, early diagnosis of FH can allow development of public health approaches to begin early treatment of FH and prevent of future cardiovascular events. In the last years, a big effort has been establishing new methodologies for assaying activity of these variants. Substitution of radioactivity for fluorescence based methodologies has lowered the cost and provided a feasible and accessible tool to characterize LDLr variants. Our group has been actively using and optimizing these fluorescent techniques to characterize and classify LDLr variants. In order to provide an accurate classification, we have also developed solid-phase immunoassays to determine LDLr binding affinity to LDL that will help to understand the phenotype of patients carrying Class 3 LDLr variants. In addition, these techniques allow the characterization of *APOB* and *APOE* pathogenic variants, as well as *PCSK9* gain and loss of function variants [75,111,112,113,114].

## Figures and Tables

**Table 1:** Summary of advantages and disadvantages of radioactive and fluorescence-based methodologies used to characterize the activity of LDLr variants.

Differences between Functional Validation Methodologies	
Radioactivity	Fluorescence
Highly reproducible	Highly reproducible
Highly sensitive activity measurements	Highly sensitive activity measurements
Stable labeling	Stable labeling
Risk of exposure to radioisotopes	Nonradioisotopes used
Ethical considerations regarding waste elimination	In combination with CLSM allow LDLr classification
Noncompatible with CLSM	

Disadvantages are shown in red.

**Table 2:** LDLr variants characterized and classified by fluorescent-based methodologies at Instituto Biofisika (UPV/EHU, CSIC) and Departamento de Bioquímica, Universidad del País Vasco.

Functional Validated and Classified LDLr Variants	Classification	LDLr Activity	Reference
c.226G>T p.(Gly76Trp)	Nonpathogenic	100%	[72]
c. 292G>A (p.Gly98Ser)	Nonpathogenic	100%	[73]
c.346T>C (p.Cys116Arg)	Class 3	25%	[74]
c.464G>A (p.Cys155Tyr)	Class 3	<20%	[69]
c.502G>A (p.Asp168Asn)	Class 3	40%	[74]
c.514G>A (p.Asp172Asn)	Class 3	<2%	[74]
c.769C>T (p.Arg257Trp)	Nonpathogenic	100%	[74]
c.806G>A (p.Gly269Asp)	Nonpathogenic	100%	[67]
c.829G>A (p.Glu277Lys)	Nonpathogenic	100%	[72]
c.862G>A (p.Glu288Lys)	Class 3	60%	[67]
c. 890A>C (p.Asn297Thr)	Nonpathogenic	100%	[73]
c.895G>A (p.Ala299Thr)	Class 3	60%	[67]
c.898A>G (p.Arg300Gly)	Class 3	60%	[74]
c.902A>G (p.Asp301Gly)	Class 3	40%	[74]
c.1216C>T (p.Arg406Trp)	Class 2b or 5	60%	[72]
c.1246C>T (p.Arg416Trp)	Class 5	60%	[69]
c.1285G>C (p.Val429Leu)	Class 2a	<10%	[70]
c.1322T>C (p.Ile441Thr)	Class 2a	<10%	[72]
c.1336 C>G (p.Leu446Val)	Nonpathogenic	100%	[75]
c.1361C>A (p.Thr454Asn)	Class 5	60%	[69]
c.1468T>C (p.Trp490Arg)	Class 2a	<10%	[70]
c.1633G>T (p.Gly545Trp)	Class 2a	<10%	[72]
c.1723G>T (p.Leu575Phe)	Class 2	60%	[76]
c.1729T>G (p.Trp577Gly)	Class 2a	<10%	[69]
c.1744C>T (p.Leu582Phe)	Class 2	60%	[76]
c.1942T >C (p.Ser648Pro)	Class 2b	<25%	[70]
c.2053C>T (p.Pro685Ser)	Class 2b	<75%	[70]
c.2475C>A (p.Asn825Lys)	Class 4	60%	[69]
c.2575G>A (p.Val859Met)	Nonpathogenic	100%	[72]

**Table 3:** ClinVar annotated LDLr variants functionally characterized ex vivo or in vitro by radioactive, fluorescence-based or other techniques.

	Ex Vivo		
Functional validated LDLr variants	LDLr activity	Method	Reference
c.1A>T (p.Met1Leu)	residual	Radioactivity	[79]
c.28T>A (p.Trp10Arg)	40%	Radioactivity	[80]
c.81C>G (p.Cys27Trp)	15–30%	Radioactivity	[20]
c.265T>C (p.Cys89Arg)	<5% Comp Htz	Radioactivity	[81]
c.268G>T (p.Asp90Tyr)	not determined	Radioactivity	[82]
c.407A>T (p.Asp136Val)	76% Htz	Fluorescence	[83]
c.418G>A (p.Glu140Lys)	30% Comp Htz	Radioactivity	[82]
c.443G>C (p.Cys148Ser)	2%	Radioactivity	[84]
c.530C>T (p.Ser177Leu)	<2%	Radioactivity	[85]
c.590G>T (p.Cys197Phe)	<2% Comp Htz	Radioactivity	[20]
c.590G>A (p.Cys197Tyr)	<2% Comp Htz	Radioactivity	[20]
c.662A>G (p.Asp221Gly)	<2% Comp Htz	Radioactivity	[20]
c.670G>A (p.Asp224Asn)	<2%	Radioactivity	[20]
c.676T>C (p.Ser226Pro)	<2%	Radioactivity	[20]
c.681C>G (p.Asp227Glu)	<2%	Radioactivity	[20]
c.682G>C (p.Glu228Gln)	2–5% Comp Htz	Radioactivity	[20]
c.796G>A (p.Asp266Asn)	<2%	Radioactivity	[84]
c.798T>A (p.Asp266Glu)	15–30%	Radioactivity	[20]
c.910G>A (p.Asp304Asn)	5–15%	Radioactivity	[20]
c.917C>T (p.Ser306Leu)	2–5% Comp Htz	Radioactivity	[20]
c.953G>A (p.Cys318Arg)	2–5%	Radioactivity	[20]
c.974G>A (p.Cys325Tyr)	<64%	Fluorescence	[62]
c.1003G>A (p.Gly335Ser)	30–40% Htz	Radioactivity	[20]
c.1013G>A (p.Cys338Tyr)	<10%	Radioactivity	[86]
c.1027G>A (p.Gly343Ser)	15–30% Comp Htz	Radioactivity	[20]
c.1055G>A (p.Cys352Tyr)	15–30% Comp htz	Radioactivity	[20]
c.1056C>G (p.Cys352Trp)	9%	Radioactivity	[81]
c.1090T>C (p.Cys364Arg)	15–30%	Radioactivity	[20]
c.1124A>G (p.Tyr375Cys)	<40%	Radioactivity	[87]
c.1135T>C (p.Cys379Arg)	15–30%	Radioactivity	[20]
c.1222G>A (p.Glu408Lys)	5–10%	Radioactivity	[88]
c.1252G>A (p.Glu418Lys)	<70 Comp Htz	Radioactivity	[89]
c.1285G>A (p.Val429Met)	<2%	Radioactivity	[90]
c.1291G>A (p.Ala431Thr)	5–15%	Radioactivity	[42]
c.1297G>C (p.Asp433His)	<10%	Radioactivity	[89]
c.1301C>A (p.Thr434Lys)	5–15% Comp Htz	Radioactivity	[20]
c.1432G>A (p.Gly478Arg)	2–5% Comp Htz	Radioactivity	[20]
c.1444G>A (p.Asp482Asn)	15% Comp Htz	Radioactivity	[88]
c.1567G>A (p.Val523Met)	15–30%	Radioac+Fluores.	[42,81]
c.1618G>A (p.Ala540Thr)	<50%	Radioactivity	[91]
c.1637G>A (p.Gly546Asp)	<2%	Radioactivity	[20]
c.1646G>A (p.Gly549Asp)	<2%	Radioactivity	[42]
c.1694G>T (p.Gly565Val)	<2%	Radioactivity	[20]
c.1702C>G (p.Leu568Val)	25%	Radioactivity	[89]
c.1729T>C (p.Trp577Arg)	<5%	Fluorescence	[92]
c.1731G>A (p.Trp577Cys)	64%	Fluorescence	[93]
c.1735G>A (p.Asp579Asn)	<2% Comp Htz	Radioactivity	[20]
c.1775G>A (p.Gly592Glu)	<5% Comp Htz	Radioactivity	[20]
c.1796T>C (p.Leu599Ser)	5–15%	Radioactivity	[20]
c.2000G>A (p.Cys667Tyr)	<2%	Radioactivity	[94]
c.2054C>T (p.Pro685Leu)	15–30%	Radioactivity	[95]
c.2177C>T (p.Thr726Ile)	15–30% Comp Htz	Fluorescence	[20]
c.2389G>T (p.Val797Leu)	not determined	Other techniques	[96]
c.2389G>A (p.Val797Met)	not determined	Other techniques	[97]
c.2479G>A (p.Val827Ile)	15–30% Comp Htz	Radioactivity	[20]
**In vitro**			
**Functional validated LDLr variants**	**LDLr activity**	**Method**	**Reference**
c.58G>A (p.Gly20Arg)	100%	Fluorescence	[98]
c.226G>T (p.Gly76Trp)	100%	Fluorescence	[72]
c.259T>G (p.Trp87Gly)	25–100%	Radioactivity	[94]
c.268G>A (p.Asp90Asn)	55%	Fluorescence	[99]
c.301G>A (p.Glu101Lys)	15–30%	Radioactivity	[100]
c.344G>A (p.Arg115His)	64%	Fluorescence	[101]
c.346T>C (p.Cys116Arg)	25%	Fluorescence	[74]
c.464G>A (p.Cys155Tyr)	<20%	Fluorescence	[74]
c.502G>A (p.Asp168Asn)	40%	Fluorescence	[74]
c.502G>C (p.Asp168His)	<2%	Radioactivity	[102]
c.514G > A (p.Asp172Asn)	40%	Fluorescence	[74]
c.589T>C (p.Cys197Arg)	<10%	fluorescence	[103]
c.665G>T (p.Cys222Phe)	33%	Fluorescence	[104]
c.769C>T (p.Arg257Trp)	100%	Fluorescence	[74]
c.782G>T (p.Cys261Phe)	<20%	Radioactivity	[105]
c.806G>A (p.Gly269Asp)	100%	Fluorescence	[67]
c.829G>A (p.Glu277Lys)	100%	Radioactivity	[106]
c.862G>A (p.Glu288Lys)	60%	Fluorescence	[67]
c.895G>A (p.Ala299Thr)	60%	Fluorescence	[67]
c.898A>G (p.Arg300Gly)	60%	Fluorescence	[74]
c.902A>G (p.Asp301Gly)	40%	Fluorescence	[74]
c.986G>A (p.Cys329Tyr)	31%	Fluorescence	[99]
c.1072T>C (p.Cys358Arg)	67–72%	Fluorescence	[93]
c.1136G>A (p.Cys379Tyr)	<40%	Radioactivity	[107]
c.1186G>A (p.Gly396Ser)	100%	Radioac+Fluores.	[108]
c.1216C>T (p.Arg406Trp)	60%	Fluorescence	[72]
c.1246C>T (p.Arg416Trp)	60%	Fluorescence	[69]
c.1268T>C (p.Ile423Thr)	54%	Radioactivity	[99]
c.1285G>C (p.Val429Leu)	<10%	Radioactivity	[71]
c.1322T>C (p.Ile441Thr)	<10%	Fluorescence	[72]
c.1361C>A (p.Thr454Asn)	60%	Fluorescence	[69]
c.1468T>C (p.Trp490Arg)	<10%	Radioactivity	[71]
c.1633G>T (p.Gly545Trp)	<10%	Fluorescence	[72]
c.1664T>C (p.Leu555Pro)	<2%	Radioactivity	[109]
c.1690A>C (p.Asn564His)	100%	Fluorescence	[110]
c.1729T>G (p.Trp577Gly)	<10%	Fluorescence	[69]
c.1744C>T (p.Leu582Phe)	60%	Fluorescence	[76]
c.1747C>T (p.His583Tyr)	<60%	Radioactivity	[108]
c.1942T>C (p.Ser648Pro)	<25%	Radioactivity	[71]
c.2053C>T (p.Pro685Ser)	<75%	Radioactivity	[71]
c.2093G>T (p.Cys698Phe)	<10%	Fluorescence	[72]
c.2396T>G (p.Leu799Arg)	residual	Other techniques	[44]
c.2475C>A (p.Asn825Lys)	60%	Fluorescence	[69]
c.2483A>G (p.Tyr828Cys)	<2% Comp Htz	Radioactivity	[33]
c.2575G>A (p.Val859Met)	100%	Radioactivity	[71]
